# Repertoire of the *Bacillus thuringiensis* Virulence Factors Unrelated to Major Classes of Protein Toxins and Its Role in Specificity of Host-Pathogen Interactions

**DOI:** 10.3390/toxins11060347

**Published:** 2019-06-17

**Authors:** Yury V. Malovichko, Anton A. Nizhnikov, Kirill S. Antonets

**Affiliations:** 1 Laboratory for Proteomics of Supra-Organismal Systems, All-Russia Research Institute for Agricultural Microbiology (ARRIAM), St. Petersburg 196608, Russia; a.nizhnikov@arriam.ru; 2 Faculty of Biology, St. Petersburg State University, St. Petersburg 199034, Russia

**Keywords:** *Bacillus thuringiensis*, *Bt*, virulence, specificity, toxin, insect, metalloprotease, chitinase, host, pathogen

## Abstract

*Bacillus thuringiensis* (*Bt*) is a Gram-positive soil bacteria that infects invertebrates, predominantly of Arthropoda phylum. Due to its immense host range *Bt* has become a leading producer of biopesticides applied both in biotechnology and agriculture. Cytotoxic effect of *Bt*, as well as its host specificity, are commonly attributed either to proteinaceous crystal parasporal toxins (Cry and Cyt) produced by bacteria in a stationary phase or to soluble toxins of Vip and Sip families secreted by vegetative cells. At the same time, numerous non-toxin virulence factors of *Bt* have been discovered, including metalloproteases, chitinases, aminopolyol antibiotics and nucleotide-mimicking moieties. These agents act at each stage of the *B. thuringiensis* invasion and contribute to cytotoxic properties of *Bt* strains enhancing toxin activity, ensuring host immune response evasion and participating in extracellular matrix degeneration. In this review we attempt to classify *Bt* virulence factors unrelated to major groups of protein toxins and discuss their putative role in the establishment of *Bt* specificity to various groups of insects.

## 1. Introduction

Gram-positive sporulating bacterium *Bacillus thuringuensis* (*Bt*) of phylum Firmicutes belongs to *Bacillus cereus* (*Bc*) group which includes an opportunistic pathogen *B. cereus* itself, as well as an obligatory pathogen *B. anthracis* [1]. In contrast to the two latter species, *Bt* does not affect vertebrates, though it shows toxicity to several mammalian cell lines [2], but rather, is referred to as an insect pathogen infecting their hosts on larval stages. However, its real host spectrum appears to comprise a much broader range of arthropods as well as nematodes of order Rhabditida, fungi, protozoans and terrestrial gastropods [3,4,5,6]. Because of its striking insecticidal activity and wide range of affected species, *Bt* is widely used either as a biopesticide [7] or as a source of resistance determinants for transgenic crops [8].

Though they can exist as free-living vegetative cells, *Bt* are usually isolated from their environment in the form of spores [9]. Once they enter the host’s organism, the spores use their immense arsenal of virulence factors to transfer from digestive organs to circulating fluids, such as blood or haemolymph, where they transit to the vegetative stage, propagate and disseminate within the host’s organism. After the host dies of a resulting septicemia, the bacteria dwelling in its cadaver propagate until they exhaust all consumable organics and then transit to sporulation. Such ecological strategy involving exploitation of both living host and its remnants is known as necromeny and can be viewed as a specific form of symbiotic interactions [10].

Insecticidal activity of *Bt* is usually attributed to the proteinaceous toxins produced at various stages of the bacterial life cycle. Vegetative *Bt* cells secrete soluble toxins comprising Vip (vegetative insecticidal proteins) and Sip (secreted insecticidal proteins) protein families. The Vip family includes four subfamilies: the Vip1 and Vip2 subfamilies comprise heterodimeric toxins, which inhibit actin polymerization and tend to affect insects of Coleoptera and Hemiptera orders [11]; the Vip3 subfamily members are putative pore-formers specific to lepidopteran hosts [12], and the last subfamily includes a sole protein Vip4 in which both mode of action and target range are still unknown [11]. The only known Sip protein, Sip1Aa, demonstrates toxicity against coleopteran larvae [13].

On the transition to sporulation, *Bt* shift to the production of insoluble δ-endotoxins. These toxins associate with auxiliary proteins to form crystal aggregates known as parasporal bodies, which are then released from the exosporium. δ-endotoxins include two families of non-selective pore-forming proteins, namely Cry (crystal) and Cyt (cytotoxic) [14], and demonstrate a wide range of affected hosts, including insects of Coleoptera, Lepidoptera, Diptera, Hymenoptera, Hemiptera and Orthoptera orders, as well as phytopathogenic nematodes and terrestrial gastropods. For most of the known δ-endotoxins, however, no suitable natural targets have been discovered so far, though some of these cryptic toxins show toxicity against species, which are unlikely to be encountered by *Bt* in natural conditions, such as parasitic nematodes [15] and trematodes [16] and a flagellar protist *Trichomonas vaginalis* [5]. Regardless of their structure and mode of action, to fulfill their cytotoxic properties all *Bt* toxins need to bind specific receptors exposed on membranes of host midgut cells. Besides, several toxins, for example, the members of Cry family, are secreted in the form of inactive protoxins requiring alkaline proteolysis mediated by the host’s digestive enzymes for activation [14].

At the same time, apart from the proteinaceous toxins, several other molecules produced by *Bt* appear to be crucial for virulence establishment and successful infection. Some of these factors show a cytotoxic effect on their own while others act as regulators of major toxins’ activity. In the present work, we focus on three classes of proteinaceous virulence factors standing apart from the canonical *Bt* toxins (that is, chitinases, zinc metalloproteases and cytolysins) and two groups of low-weight moieties (aminopolyol antibiotics and β-exotoxins). Here, we provide a comprehensive review of rapidly accumulating data on the virulence factors of *Bt* unrelated to major groups of protein toxins and discuss their impact on virulence and pathogenesis to elucidate their role in *Bt* host-specificity.

## 2. Proteinaceous Virulence factors of *Bt*

### 2.1. Bt Chitinases

The first barrier, which *Bt* spores ingested by insects need to overcome, is typically presented by a peritrophic membrane constituting a dense film consisting of chitin fibrils cross-linked by chitin-binding proteins called peritrophins [17]. This structure isolates apical surface of midgut epitheliocytes from the ingested nutriments thus providing protection from both mechanical damage and pathogen absorption. Depending on their content and biogenesis, peritrophic structures fall into two distinct types. Type I membranes are temporary structures formed directly around food lumps being digested. In their turn, type II membranes persist being attached to the midgut walls in its anterior section. While type I peritrophic membranes are common for the species of the orders Blattodea, Orthoptera, Coleoptera, Hymenoptera and Diptera, occurrence of type II is restricted within orders Diptera and Lepidoptera. The sanguivorous members of order Diptera, such as black flies (Diptera: Simuliidae) and mosquitoes (Diptera: Culicidae) can switch from type II to type I during metamorphosis due to transition from solid diet of larvae to the liquid feeding of mature species [18,19]. Besides solid peritrophic membranes, several insects produce a semi-liquid peritrophic gel devoid of chitin component which can sustain a gel-like structure due to a specific peritrophin and mucin content [17]. Such strategy is exploited by bean weevils (Coleoptera: Bruchidae) who feed upon legume seeds known to store lectins [20]. In species of orders Hemiptera and Thysanoptera, the peritrophic compartment is represented by a glycoprotein structure known as the perimicrovillar membrane [21]. Despite these examples of the elimination of the chitin component from peritrophic structures, complete absence of peritrophic structure rarely occurs in insects and is usually related to a low-weight molecular diet, as in the case of several hymenopteran and lepidopteran species [22].

A widely used way to penetrate these chitin-based membranes is the production of chitinases, hydrolytic enzymes capable of digesting chitins, a group of N-acetyl D-glucosamine polymers, via hydrolysis of β-(1→4)-glycoside bonds [23]. Based on their mode of action, chitinases are categorized in endochitinases, which split chitin by random internal sites resulting in the formation of di-acetylchitobiose, chitotriose, and chitotetraose, and exochitinases cleaving short chitooligosaccharides from molecule termini [24]. Chitinase substrate affinity is provided by the presence of special chitin-binding modules, or CBMs. These modules are marked by impressive structural variety, but in all cases, their chitin-binding affinity is associated with the conservative tryptophan residues [25]. In terms of glycoside hydrolase (GH) structural classification chitinases belong to three families, of which families GH18 and GH19 appear to be the largest ones and comprise both endo- and exochitinases (according to Carbohydrate-active Enzymes Database (CAZy) on 2019) [26]. Proteins belonging to these two families have been encountered in all major groups of living organisms as well as several groups of viruses [24,27].

The presence of the chitinase-encoding genes in bacterial genomes, including those of *Bacillus* isolates, allows them to assimilate chitin effectively and even use it as a sole carbon source [28,29]. Chitinases found in *Bt* belong to the GH18 family [24]. Though most of them, such as ChiA, ChiB and ChiC, act in endochitinolytic manner, occurrences of exochitinases have also been reported [30,31]. Chitinase-producing *Bt* strains often possess fungicidal activity [4] and are capable of inhibiting growth of the phytopathogenic fungi which makes them a promising antimycotic agent for agricultural needs [32,33]. Unlike their fungicidal properties, the role of chitinases in virulence of insecticidal strains of *Bt* has long been confined to the facilitation of the cytotoxicity of crystal toxins [34]. Nonetheless, several insect endoparasites such as protists of genera *Plasmodium* [35] and *Leishmania* [36], nematode *Brugia malayi* [37] and baculoviruses [38], use chitinases to digest the peritrophic membrane enveloping the lumps of ingested substrates once they get in the midgut. Since *Bt* infect their hosts mostly, and apparently exclusively, through the digestive tract, it seems plausible that they might exploit their chitinase repertoire in a similar manner.

At present, the role of chitinases in *Bt* virulence is mainly perceived through the hydrolysis of the peritrophic membranes. In most cases, chitinases act synergistically with crystal toxins and other *Bt* virulence factors. For instance, a joint inoculation of Cry-producing *Bt* ser. *israelensis* and avirulent chitinase producers bolsters the effect of crystal toxins on *Spodoptera exigua* (Lepidoptera: Noctuidae) by 2.35 times [39]. In other experiments the presence of external chitinases doubled the toxicity of Cry4Aa, Cry4Ba, Cry11Aa and Cyt1Aa against *Aedes aegyptii* (Diptera: Culicidae) [40] and enhanced the toxicity of CryIC against *S. littoralis* by six times [41]. Also, enhancement of Cry4Ba toxicity against its natural targets *A. aegyptii* and *Culex quinquefasciatus* (Diptera: Culicidae) in the presence of chitin-binding agent calcofluor white may serve as an indirect evidence for the cumulative effect of Cry and chitinases or any other chitin-binding moieties [42]. Notably, there is at least one example of analogous synergy of chitinases and unidentified Vip toxin [30] which suggests that not only the Cry family but also other major classes of *Bt* toxins benefit in their cytotoxicity on the chitinase production background.

The diversity of insect peritrophic structures suggests that chitinases may be involved in the control of the host-specificity of *Bt*. For instance, CBM-containing chitinases mostly split crystal chitin, also known as α-chitin, while specificity of other chitinase families may differ [43]. Besides canonical chitinases, a unique CBM2-containing chitinase BthChi74 was shown to bind not only crystal chitin but also cellulose [44]. Apart from their substrate affinity, the CBM structure may be affected by the temperature and pH values, since these two factors in the midgut compartment differ between insect species depending on their phylogenetic position, diet, ontogenesis stage and gut microflora diversity [45,46].

Finally, it is worth mentioning that several species of bacterial entomopathogens, such as orthopteran pathogens of *Sanguibacter* genus [47] and Gram-negative bacterium *Yersinia entomophaga* known to infect *Costelytra zealandica* (Coleoptera: Scarabaeidae) [48], exploit chitinases as their main virulence factors. Considering that chitinase-producing *Bt* strains have been tested mainly on the insect species sharing common structure of peritrophic matrix, it is possible that the role of chitinases in *Bt* virulence and host specificity needs additional clarification and may be utterly underestimated.

### 2.2. Bt Metalloproteases

After penetrating the chitin-rich peritrophic membrane, *Bt* cells need to overcome several barriers enriched with different protein factors, such as a mucin layer, a basal lamina and cadherins. To achieve this aim, *Bt* utilize many different metalloproteases. By definition, these proteins represent enzymes capable of hydrolyzing peptide bond in the presence of one or more metal ions, usually those of zinc (II) [49]. One of the existing classifications divides all known metalloproteases into two subclasses depending on the presence of one or two metal ions in the catalytic site, which subsequently diverge into tribes, clans and, finally, distinct families [50]. Despite their structural diversity, monometallic metalloproteases share an ordered single-displacement mechanism of hydrolysis catalyzed by a metal ion and a neutral or basic residue within the active center, and operate in the common milieu, such as pH = 6.5 [50].

Based on the structure of metal-binding motifs, monometallic zinc metalloproteases are divided into five tribes, of which the zincin tribe distinguished by the HEXXH consensus metal-binding motif is the largest one [51]. In turn, zincins split into five clans according to an active residue of a loop adjacent to a metal-binding motif, namely metzincins, gluzincinz, aspzincins, S2P-zincins and FtsH-like metalloproteases. Finally, notation suggested by the MEROPS database (https://www.ebi.ac.uk/merops/index.shtml) divides all known metalloproteases into 103 families with each family name comprising of a letter M standing for ‘metalloprotease’ and a respective family number [52]. Due to their structural and functional variability metalloproteases serve as virulence factors in both Gram-positive and Gram-negative bacteria as well as pathogenic fungi and protists [49]. Their insecticidal properties alongside with other proteases have been reviewed previously in [53]. According to MEROPS, 30 metalloprotease families have been encountered in *Bt* including the most important M60, M6, M9 and M73. Here we summarize data on these metalloprotease families known to impact pathogenicity of *Bt* at different stages of infection.

#### 2.2.1. Enhancin-Like Metalloproteases

Enhancin is a zinc metalloprotease of *Trichoplusia ni* granulovirus (TnGV) marked with M60 family metalloprotease domain [54]. In viruses, enhancins serve mostly to digest the peritrophic matrix in order to facilitate virion transition to haemocoel [55]. Among the components of the peritrophic matrix, invertebrate intestintal mucin (IIM) appears to be the primary target of enhancin-mediated proteolysis. This protein was first discovered in *T. ni* (Lepidoptera: Noctuidae) larvae, though its orthologues are present in the species outside Lepidoptera order, such as *T. castaneum* (Coleoptera: Tenebrionidae) [56]. Viral enhancins were shown to positively affect *Bt* virulence against at least six lepidopteran species: *T. ni*, *H. zea*, *H. virescens*, *S. exigua*, *Chrysodeixis includens* and *Anticarsia gemmatalis* (Lepidoptera: Noctuidae) [57]. Since then, genes encoding enhancin-like proteins have been found in several bacterial genomes, while in *B. cereus* and *B. thuringiensis* these genes were shown to belong to PlcR (Phospholipase C Regulator) regulon, whose activity is triggered in the end of the phase of exponential growth and is strongly mitigated during the stationary stage shift due to the general sporulation regulator Spo0A [58,59,60]. These genes are called *bel* (for *Bacillus* enhancin-like).

The first *bel*-encoded protein discovered, mpbE (metalloprotease *Bacillus* enhancin), from *B. cereus* strain ATCC14579 failed to show any virulence reinforcement in *G. mellonella* larvae assays [60]. However, the Bel protein isolated from strains YBT1520 and BMB171 in 2009 was found to directly influence *Bt* virulence against *H. armigera*: the knockout of the *bel* gene led to rise of *Bt* semilethal dose by 5.8 times, while the addition of Bel to a purified Cry1Ac preparation bolstered its lethality from 34.2 to 74.4% [61]. Bel shares 20–30% sequence identity with TnGV enhancins and operates in the similar manner hydrolyzing IIM. For now, at least one more *Bt* enhancin-like protein has been described [62]; although it shares 23–41% identity with viral enhancins, it, however, did not show any toxicity to *S. exigua* and *T. ni* larvae in respective assays.

#### 2.2.2. InhA Metalloproteases

Another example of PlcR-regulated *Bt* metalloproteases is an InhA group of family M6 [63] represented by three proteins with similar functions [64]. All three proteins participate in numerous processes associated with different stages of *Bt* infection. For instance, InhA1 plays an ambiguous role in *Bt* infection. On the one hand, it was shown that InhA1 is capable of hydrolyzing cecropins and attacins, antimicrobial peptides playing the key role in the insect humoral immune response [65]. Beyond that, the exosporal localization of InhA1 molecules in those *Bc* species virulent to vertebrates allows them to escape phagocytosis by macrophages by cleaving membrane-associated proteins [66]. This function can be extrapolated on *Bt* InhA1 proteins since insects were clearly shown to possess macrophage-like professional phagocytic cells [67]. On the other hand, the proteolytic activity of InhA1 resolves in the destruction of host extracellular matrix, including basal lamina of midgut epithelium, which is essential for haemolymph infesting [65]. InhA2 and InhA3 bear similar functions to those of InhA1 and share 66% and 72% sequence identity with it, respectively [64]. However, in contrast to InhA1, InhA2 does not affect *Bt* virulence in the absence of other PlcR regulon products [68]. In its turn, InhA3 is incapable of cecropin proteolysis, though its general proteolytic properties are preserved [64].

In the haemocoel, InhA1 demonstrates a strong toxic effect, which can be explained by non-specific proteolysis of haemolymph components [69]. Cytotoxic effect of InhA1 was first demonstrated in oral infection assays of *Spodoptera littoralis* (Lepidoptera: Noctuidae) larvae [70]. This effect is manifested through detachment of epitheliocytes from basal lamina due to the lysis of its main components (such as type IV collagen, laminin and fibronectin) as well as partial cell lysis. The same research provided evidence for the cumulative effect of InhA1 *Bt* crystal toxins. Similar enhancement of cytotoxicity was shown for joint application of Cry1C and InhA2 against *Galleria mellonella* (Lepidoptera: Pyralidae) larvae [71].

#### 2.2.3. Other *Bt* Metalloproteases and Their Association with Biofilm Formation

Recently a new *Bt* metalloprotease, ColB, has been discovered [72]. ColB belongs to M9, or collagenase, family, which comprises numerous bacteria virulence factors [73]. Presence of ColB drastically enhances the cytolytic activity of several Cry toxins, including Cry5Ba, Cry55Aa and Cry6Aa, against *H. armigera* and nematodes *Caenorhabditis elegans*. ColB acts through proteolysis of both E-cadherins comprising epithelial intercellular contacts and collagens of basal lamina, resulting in degradation of midgut epithelium [74]. Thus, ColB mediates *Bt* transition from digestive tract to host haemocoel and subsequent spreading throughout the host body. Similar to that of ColB is the effect of Bmp1 M4-family protease, which also belongs to PlcR regulon, even though its involvement in *Bt* virulence has been shown for nematicidal strains only [75].

A casein-cleaving membrane protease (CCMP), or camelysin (CalY), poses another example of the *Bt* neutral metalloproateases [76]. According to the MEROPS classification, CalY belongs to M73 metalloprotease family. Although CalY demonstrates proteolytic activity against a broad range of proteins, including casein, actin and collagen [77], it was also shown to co-participate in biofilm formation with TasA fibril-forming protein [78]. Biofilms are bacterial communities floating on the culture medium or sticking to solid surfaces with the help to strong extracellular matrix, which consists of exopolysaccharides, proteins and extracellular DNA [79]. Interestingly, the proteinaceous component of such matrix tends to form amyloids, extremely stable fibrils with highly ordered spatial structure [80,81], in many different bacteria, such as *Bacillus* [82] and *Escherichia coli* [83]. TasA fibrils were previously reported to possess amyloidogenic properties based on their structure and detergent resistance [84], but were not tested for their biological activity in amyloid-like state, thus remaining the status of TasA as a functional amyloid dubious [85]. Biofilm formation is an important stage of attachment to the host tissues for many pathogens [86]. CalY plays the key role in both processes, in the adhesion to insect cells and in the biofilm formation, which makes it a very important virulence factor in *Bt* [78]. Interestingly, the evolutionary conserved M60 zinc metalloprotease domain found in both Gram-positive and Gram-negative bacteria [87], was found to be amyloidogenic as well [88,89] also providing a link between bacterial metalloprotease virulence factors and amyloid formation.

Apart from the involvement in the biofilm formation, CalY was shown to increase the cytolytic properties of Cyt1Aa (from 40 to 70%) and Cyt2Ba (from 6% to 50%) protoxins in rabbit erythrocyte assays [90]. Though a native proteolytic activation of Cry toxins by host proteases is realized at the alkaline values of pH, in the presence of CalY toxins they turn into active state at pH = 6.5. Thus, the synergistic effect of crystal toxins and metalloproteases might lie not only in a similar influence on epithelial components, but also in the formation of host-independent mechanism of the protoxin activation [90,91].

#### 2.2.4. Role of Metalloproteases in Processing of Cry-Toxins

Judging by an example of CalY, it seems possible that *Bt* metalloproteases can hydrolyze not only host proteins but also bacterium’s own toxins. Such host-independent toxin proteolysis has already been found in other bacteria, for instance, in *Vibiro cholera,* which produces metalloproteases activating hemolysins [92]. Though *Bt* toxin can be activated by trypsic enzymes of various origin, cases of alteration of toxin’s host range by application of gut juice from different hosts have been reported [93]. The most intriguing examples come from the hemipterans who exploit membrane-bound cysteine proteases rather than serine proteases for protein digestion. For instance, in the pea aphid *Acyrthosiphon pisum* native gut proteases fail to cleave Cry3Aa sufficiently, which resolves in partial activation of the toxin [94]. At the same time, pretreatment by serine proteases restored toxicity of Cry3A, Cry4A and Cry11A in the same species [95]. This suggests that impaired proteolysis might be a reason for low susceptibility of hemipterans to *Bt* toxins though it might be affected by many other factors [96]. The assumption of *Bt* metalloprotease-mediated toxin processing in this case is further underpinned by the lower rates of pH in the aphid anterior midgut which are quite close to optimal for metalloprotease activity [97]. Thus, metalloproteases may act, not only against host proteins, but also provide host-independent systems for toxins activation.

### 2.3. Cytolysins

Formerly known as *B. sphaericus*, *Lysinibacillus sphaericus*, or *Ls*, is another entomopathogenic representative of Firmicutes phylum [98]. *Ls* is mostly known to infect mosquitoes (Diptera: Culicidae) by means of production of proteinaceous toxins, such as non-canonical Cry toxins Cry48Aa1/Cry49Aa1 and binary toxins BinA/BinB [14] Mtx vegetative toxins [99] and several specific cytolysins such as sphaericolysin [100]. A related toxin named alveolysin, was initially discovered as a virulence factor of *Paenibacillus alvei* (previously *B. alvei*), another species close to the *Bacillus* genus [101].

Both sphaericolysin and alveolysin belong to the thiol-activated cytolysin (TACY) family of cytolysins, a class of toxins, which act through formation of Na^+^-conductive olygomeric pores with subsequent cell swelling and lysis [102,103]. These cytolysins often serve as virulence factors of Gram-positive bacteria [102], while their occurrence in Gram-negative bacteria seems rare [104]. Since TACYs exploit membrane cholesterol as a main receptor, it is likely that these toxins may embrace a wide range of the affected species [102]. Indeed, sphaericolysin was shown to affect two such phylogenetically distant species as *Blattella germanica* (Blattodea: Ectobiidae) and *Spodoptera litura* (Lepidoptera: Noctuidae) [100]. In *Bt*, several cytolysins discovered so far bear a close structural resemblance to the *Ls* toxins, which suggests a similar mode of action and the range of affected hosts [14]. This idea is supported by observation of pathogenicity of several *Bt* strains to species of order Blattodea despite the absence of known Blattodea-specific *Bt* toxins [100,105].

## 3. Non-Proteinaceous Virulence Factors of *Bt*

### 3.1. Zwittermycin A

Produced by several strains of *B. thuringiensis* and *B. subtilis*, zwittermycin A (ZwA) is the only known antibiotic moiety of aminopolyol nature [106]. Structurally ZwA constitutes a peptide decorated with accessory amino and hydroxyl groups and shows resemblance to poliketide antibiotics. The biosynthesis of zwittermycin A is controlled by nine open reading frames forming a 16 kb gene cluster; proteins encoded within these frames form a unitary macromolecular complex which operates all stages of the antibiotic synthesis including the extraribosomal formation of peptide bonds [107]. Inactivation of ZwA, which forms the main mechanism of *Bt* autoresistance to its own antibiotic, is mediated by acetylase encoded by the *zmaR* gene associated with the synthetic cluster [108]. ZwA affects a wide spectrum of bacterial species as well as some eukaryotes including phytopathogenic fungi and oomycetes [109]. However, its mode of action remains elusive. Though resistant strains of *E. coli* are known to possess mutations in *rpoB* and *rpoC* genes encoding RNA polymerase core subunits, no direct effect of ZwA on transcription or any other stage of nucleic acid metabolism has been shown so far [110].

Despite the lack of obvious toxicity for eukaryotes, ZwA somehow appears to enhance *Bt* virulence. In particular, the synergistic effect of ZwA and main virulence determinants was demonstrated for lepidopteran-specific strains belonging to *kurstaki* serovar. The increase of ZwA production by site-specific mutagenesis enhanced the insecticidal effect of the respective strain against *S. exigua* and *H. armigera* (Lepidoptera: Noctuidae) by 115.4 and 25.9%, respectively [111]. Alternatively, a cooperative inoculation of *Bt* and avirulent *B. cereus* ZwA-producing strain resulted in the burst of *Bt* pathogenicity against *Lymantria dispar* (Lepidoptera: Erebidae) [112]. Such synergy between zwittermycin A and proteinaceous toxins is likely to be related to the eradicating effect of ZwA on midgut microbiota. According to one of the hypotheses present, these bacteria can influence pH rate in the midgut, which in its turn alleviates toxin solubility and proteolysis [113,114].

### 3.2. β-Exotoxins

Low weight toxins produced by some of the *Bt* strains vegetative cells are commonly referred to as β-exotoxins [115]. Due to preservance of their biocidal properties after being exposed to high temperature β-exotoxins are sometimes called thermostable *Bt* exotoxins which emphasizes their difference from thermolabile proteinaceous exotoxins. The most widespread moiety of this class is thuringiensin, or Thu, also called type I β-exotoxin [116]. Thuringiensin constitutes an adenosine analog whose 5′-hydroxile is conjugated with a phosphorylated diglucuronic acid residue. Type II β-exotoxin, having been discovered later, presumably constitutes a similar analog of uridine [117]. Thu biosynthesis is operated by *thu* cluster consisting of eleven genes which is usually located on large plasmids [118]. It is noteworthy that both the ability of Thu production and amount of toxin produced correlate with the presence of particular *cry* genes and serovar identity of the strain [119,120].

Cytotoxic activity of thuringiensin is likely to be a consequence of its structural similarity to ATP, since it is capable of binding to ATP-binding sites of RNA polymerase causing transcription inhibition [121,122]. The morphological effect of Thu on larval midgut epithelium was demonstrated on dipteran *Culex sitiens* and includes microvilli reduction and fragmentation of cellular membranous compartments such as granular ER and Golgi apparatus [123]. Because of the conservative structure of eukaryotic RNA polymerase functional sites β-exotoxins affect an outstandingly wide range of insect hosts from different orders. To date, insecticidal effect of thuriniensin has been shown for *Anastrepha ludens*, *A. obliqua* and *A. serpentina* (Diptera: Tephritidae) [124], *C. sitiens* (Diptera: Culicidae) [123], *Lasioderma serricorne* (Coleoptera: Anobiidae) [125], *H. armigera*, *H. zea*, *S. exigua*, *Heliothis virescens*, *T. ni* (Lepidoptera: Noctuidae) [116,126], *Estigmene acrea* (Lepidoptera: Erebidae), *Pectinophora gossypiella* (Lepidoptera: Gelechiidae) [126], *Leptinotarsa decemlineata* (Coleoptera: Chrysomelidae) [127], *Anthonomus grandis* (Coleoptera: Curculionidae) [103], *Lygus hesperus* (Hemiptera: Miridae) [128]; some sources also state thuringiensin toxicity for Orthoptera and Neuroptera [116]. Apart from insects, Thu is toxic to mites of the Tetranychidae family [129] and nematodes of order Rhabditida [130]. In addition, thuringiensin is potentially harmful for mammals as it was shown to cause inflammatory processes leading to lung tissue damage because of adenylate cyclase activation [131].

## 4. Discussion

**Figure 1:**
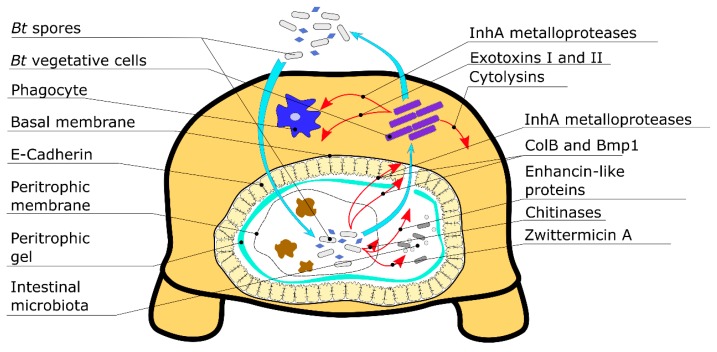
*Bt* minor virulence factors produced on different stages of its life cycle and their respective targets. The figure depicts a schematic cross-section of an insect larva body; denoted are the *Bt* virulence factors and their modes of actions.

Taken together, apart from the major proteinaceous toxins *B. thuringiensis* produces a vast range of molecules affecting its pathogenesis which can be perceived as virulence factors. Moreover, these molecules not only enhance toxicity formed by crystal toxins but also exploit principally different mechanisms of pathogenesis that are summarized in Table 1 and Figure 1. These factors can be divided into two major groups. The first group contains proteinaceous factors showing certain host specificity, while the second group is formed by low weight moieties, which, vice versa, affect a wide range of organisms in a non-specific way.

Transition from the digestive tract to host body cavities, such as haemocoel in the case of insect hosts, is the key stage of *Bt* infection (Figure 1). At this stage numerous virulence factors, such as Cry toxins, chitinases and metalloproteases are resolved to penetrate midgut walls. Since all of these factors show, at least to some degree, specificity in their hosts affected, this transition may be the most principal step to define bacterium’s host range, and minor virulence factors here may be viewed as a mean of its enlargement. Another crucial function of minor virulence factors is the alleviation of the host’s immune response which takes place mostly in the haemocoel. This process recruits both specific determinants, such as InhA metalloproteases, and unspecific factors defined by low diversity and functional degeneracy (Figure 1). That is, thuringiensin is shown to be toxic for several phylogenetically distant insect orders as well as several other groups of invertebrates and even vertebrates [121] (Table 1). Since the structure of β-exotoxins potentially inhibits all the ATP-dependent enzymes, such a wide range of vulnerable species does not seem to be surprising. A similar broad range of susceptible species can be expected from other low molecular inhibitors; for instance, *trans*-aconitate, a Krebs cycle inhibiting agent newly discovered in *Bt*, performs in a non-specific manner and affects both nematodes and the brown planthopper *Nilaparvata lugens* (Hemiptera: Delphacidae) [132].

To estimate the spreading of the minor virulence factors across known *Bt* strains we searched for several of the discussed proteins in genome annotations available at NCBI Assembly (downloaded on 20.04.2019). Of 511 genome assemblies, we chose 468 that had annotations which comprised more than 4500 genes, and then discarded one annotation which did not contain any functional information on its entries. The results are presented in Table 2.

Unsurprisingly, chitinases seem to be present in all the analyzed strains except for one, thus suggesting that chitinases are essential for efficient *Bt* infection. This idea is sustained by the study of *chi71A* knockouts in *Bt* ser. *pakistani* which exhibited a dramatic loss of toxicity against *A. aegyptii* [133]. The only chitinase-less strain encompassed by our survey is HD73 belonging to *kurstaki* serovar, a standard crystaliferous strain notable for its modest chitinolytic activity and often used in recombinant chitinase genes assay [134]. Thus, chitinases in the broad sense pose as crucial and apparently non-specific virulence factors. To clarify their true role in host specificity, a thorough analysis of relations between specific chitinase groups and host range of a possessing strains is required as these proteins might differ in their optimal milieu. For instance, a recently discovered chitinase ChiA74 from *Bt* serovar *kenyae* strain LBIT-82 demonstrates an unusual bimodal distribution of pH optimum [135]. Such a mode of action might be a preadaptation to different environments and, thus, broaden a host range of a possessing strain.

The role of metalloproteases in determining host-specificity seems to be very diverse, because they act at different stages of pathogenesis. Proteolysis of mucin layer facilitates the penetration of peritrophic structures and epithelium exposure implying functional similarity between enhancin-like metalloproteases and *Bt* chitinases. The selective effect of the enhancin-like enzymes tied exclusively to lepidopteran hosts suggests differences in proteinaceous content of peritrophic membrane to be one of the specificity factors of *Bt* relating their metalloprotease repertoire [136]. In this light, a wide distribution of enhancins and enhancin-related metalloproteases whose genes are encountered in 59.6% of the analyzed strains seems quite bewildering. Because of the lack of information on host specificity or even serovar attribution for most of the strains deposited on NCBI Assembly verification of correlation between enhancin occurrence and anti-lepidopteran potency falls from the scope of this review. Nevertheless, of eight strains explicitly attributed to *kurstaki* serovar known for its high toxicity against Lepidoptera and Diptera [3], seven possess enhancin genes, while none were found in four strains identified as serovar *israelensis* specimens, which is known to act against dipteran species [137]. On the contrary, *Bt* matrix metalloproteases and collagenases demonstrate low specificity between hosts, presumably because of common structure of basal lamina; for instance, ColB metalloprotease equally contributes to infection of such phylogenetically distant hosts as insects and nematodes [72]. This idea is further supported by wide distribution of collagenase genes in *Bt* (Table 2).

Among *Bt* metalloproteases, InhA proteins appear to serve most various and complex functions. Apart from extracellular matrix lysis, these proteins are involved in the evasion of the humoral and, most likely, cellular immune response from the host organism. Differences in proteolytic affinity between InhA1 and InhA3 paralogues may be explained either by structural differences between metalloproteases themselves or by variety of antimicrobial peptides produced by insects. *inhA* genes are present in 59 strains in our brief survey. Interestingly, six of the eight discussed *kurstaki* strains possess these genes. An uneven distribution of different metalloproteases might by associated with the host specificity of *Bt* strains, and a more detailed assessment of their role in the establishment of host specificity including a parallel consideration of proteinaceous toxins, minor factors and the known host range of the analyzed strains might shed light on their role. At the same time, such analysis seems to be very difficult due to lacking metadata for some *Bt* strains.

In connection with metalloproteases, a probable role of amyloid fibril formation in *Bt* virulence and host-specificity seems to be very interesting. CalY, which was discovered as metalloprotease [77], was shown to form amyloid-like fibrils [78,82]. In Assembly-provided strains, *calY* is found in 12.6% of the strains, and in at least seven cases it is accompanied by *tasA*, a gene encoding another fibril-forming protein in *Bt*. Utilizing amyloid protein as a structural element of biofilm matrix is a very conservative feature shared by many different bacterial species [138]. Thus, one may expect that amyloid formation in biofilms involving co-polymerization of different virulence factors such as metalloproteases represents a general non-specific way of bacterial pathogenesis. Such amyloid formation by proteinaceous virulence factors could be important to provide their survival in the aggressive internal environment of the host body.

Recently, it was shown, that M60-like metalloprotease of *E. coli,* YghJ, forms amyloid fibrils [88,89]. YghJ is a metalloprotease involved in mucin degradation in mammalian intestine and belongs to the same family as enhancin-like enzymes, which act very host-specific. So, the role of amyloidogenesis in host specificity of *Bt* remains unclear.

Compared to other aforementioned factors, zwittermicin A shows a truly unique mode of virulence modulation. Despite its insecticidal effect has not yet been shown on other hosts rather than lepidopterans, one might suggest that similar ZwA-mediated suppression of native midgut microbiota may take place in *Bt* infection of various insects [98,99]. This, however, raises a question whether ZwA is a *bona fide* virulence factor rather than an allelopatic agent used to compete for ecological niches, both environmental and endogenous, with other microorganisms. If the latter is true, ZwA should be put in one row with other *Bt* antibiotics, such as, for example, peptides of bacteriocin group [139].

To conclude, the acquired data on the molecular mechanisms underlying the mode of action and specificity of the *Bt* virulence factors unrelated to major classes of protein toxins demonstrate their unequivocal role during *Bt* infection, state that proteinaceous factors have a greater impact on specificity of such interactions than low-weight non-protein molecules bearing a more generalized effect, and suggest involvement of functional protein aggregation in host-pathogen interactions.

## Figures and Tables

**Table 1:** The virulence factors of *Bt* unrelated to major protein toxins.

Virulence Factor			Principle of Action	Putative Targets of Virulence Modulation	Known Examples of Virulence Modulation	References *
**Group I: proteinaceous factors**	**Chitinases**		Peritrophic matrix permeabilization	Enzyme’s structure, stability and substrate affinity Structure of host’s peritrophic matrix	**Diptera** -Culicidae **Lepidoptera**- Noctuidae	[39,40,41,42]
	**Metalloproteases**	**InhA**	Antimicrobial peptide cleavage Immune response evasion Cytolysis of epithelial cells Basal membrane lysis	Structure of host’s antimicrobial moieties Proteinaceous content of basal membranes	**Lepidoptera** -Noctuiade	[65,68,69,70,71]
		**Enhancin-like proteins**	Cleavage of peritrophic mucins	IIM structure	**Lepidoptera** -Noctuidae	[61,62]
		**ColB and Bmp1**	Basal lamina lysis E-cadherin cleavage	Proteinaceous content of basal membranes Host’s E-cadherin structure	**Lepidoptera** -Noctuidae	[72,74,75]
		**CalY**	Lysis of basal lamina and extracellular matrix Biofilm formation Cry toxin processing (?)	N/A **	N/A	[76,77,84,90,91]
	**Cytolysins**		General cytotoxicity (pore formation)	Membrane steroid content; apparently, presence of receptor proteins	**Blattodea** -Ectobiidae **Lepidoptera** -Noctuidae	[98,100]
**Group II: low molecular weight factors**	**Zwittermycin A**		Gut microbiota eradication	Midgut microbial diversity	**Lepidoptera** -Erebidae- Noctuidae	[106,111,112,113]
	***β-exotoxins***		Nuclear transcription suppression	Apparently absent	**Diptera** -Tephritidae -Culicidae **Coleoptera** -Chrysomelidae -Curculeonidae **Hemiptera** **Lepidoptera** -Gelechidae -Erebidae -Noctuidae **Orthoptera**	[116,124,125,126,127,128,129,130]

* Links are given for key papers providing evidence for presence of respective factors in Bt. ** No adequate speculations can be made due to the lack of data.

**Table 2:** Distribution of proteinacious virulence factors among 467 sequenced strains.

Virulence Factor	Number of Occurrences	Percentage of Occurrences
Chitinases (various)	466	99.8
Enhancins and enhancin-like metalloproteases	276	59.1
ColB and other collagenases	461	98.7
InhA metalloproteases	59	12.6
ColY metalloprotease	59	12.6
ColY + TasA	7	1.5
